# Nanofiltration Membranes for the Removal of Heavy Metals from Aqueous Solutions: Preparations and Applications

**DOI:** 10.3390/membranes13090789

**Published:** 2023-09-12

**Authors:** Alaa El Din Mahmoud, Esraa Mostafa

**Affiliations:** 1 Environmental Sciences Department, Faculty of Science, Alexandria University, Alexandria 21511, Egypt; 2 Green Technology Group, Faculty of Science, Alexandria University, Alexandria 21511, Egypt

**Keywords:** phase inversion, nanomaterials, nano-embedded membranes, metal ions, antifouling, commercial membranes

## Abstract

Water shortages are one of the problems caused by global industrialization, with most wastewater discharged without proper treatment, leading to contamination and limited clean water supply. Therefore, it is important to identify alternative water sources because many concerns are directed toward sustainable water treatment processes. Nanofiltration membrane technology is a membrane integrated with nanoscale particle size and is a superior technique for heavy metal removal in the treatment of polluted water. The fabrication of nanofiltration membranes involves phase inversion and interfacial polymerization. This review provides a comprehensive outline of how nanoparticles can effectively enhance the fabrication, separation potential, and efficiency of NF membranes. Nanoparticles take the form of nanofillers, nanoembedded membranes, and nanocomposites to give multiple approaches to the enhancement of the NF membrane’s performance. This could significantly improve selectivity, fouling resistance, water flux, porosity, roughness, and rejection. Nanofillers can form nanoembedded membranes and thin films through various processes such as in situ polymerization, layer-by-layer assembly, blending, coating, and embedding. We discussed the operational conditions, such as pH, temperature, concentration of the feed solution, and pressure. The mitigation strategies for fouling resistance are also highlighted. Recent developments in commercial nanofiltration membranes have also been highlighted.

## 1. Introduction

**Figure 1:**
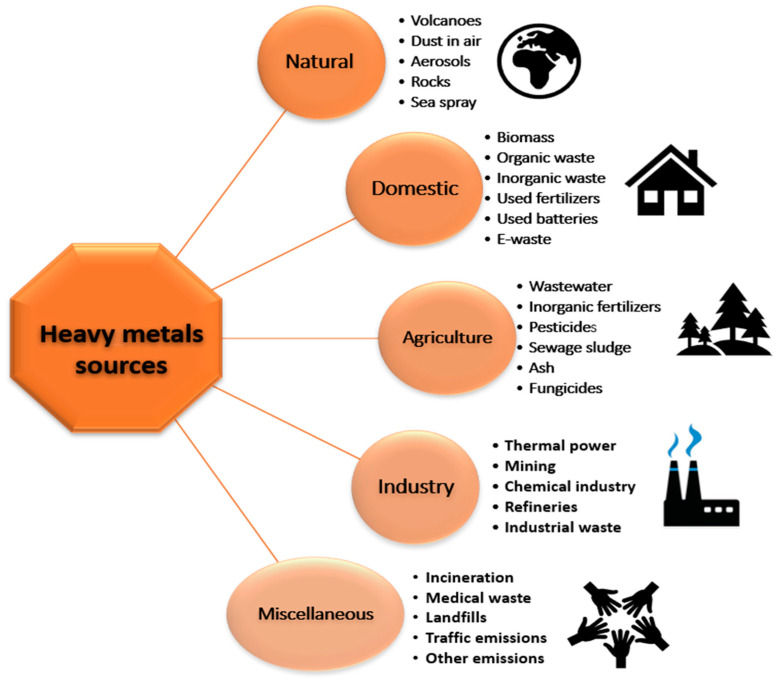
Summary of potential sources of heavy metals from natural and anthropogenic sources.

Water shortages are a problem caused by global industrialization and increasing population. Freshwater scarcity is a catastrophe in arid and semiarid areas that affects social and economic development [1]. The release of wastes and effluents into the environment has many effects such as leaching, eutrophication, pathogen spreading, and escalation of waterborne diseases [2,3]. In developing countries, most wastewater discharge without proper treatment leads to groundwater contamination and a limited clean water supply. The major contaminants are heavy metals with a high molecular weight and density of >5 g cm^−3^ compared to water [1,3]. These metals are released into the environment from natural and anthropogenic sources. Figure 1 provides a summary of the major sources of the heavy metal sources. Sources are classified based on their origin as natural, domestic, agricultural, industial, and miscellaneous sources such as incineration, medical waste, landfills, and traffic emissions [4].

Heavy metals detected in drinking water can endanger the health of people in drinking water. Although the bioaccumulation of heavy metals in humans (for instance, in lipids and the digestive system) could result in cancer and other diseases, certain heavy metals are largely exposed to populations through drinking water [5,6]. Therefore, it is vital to identify alternative water sources because many concerns are directed toward sustainable water treatment processes.

**Figure 2:**
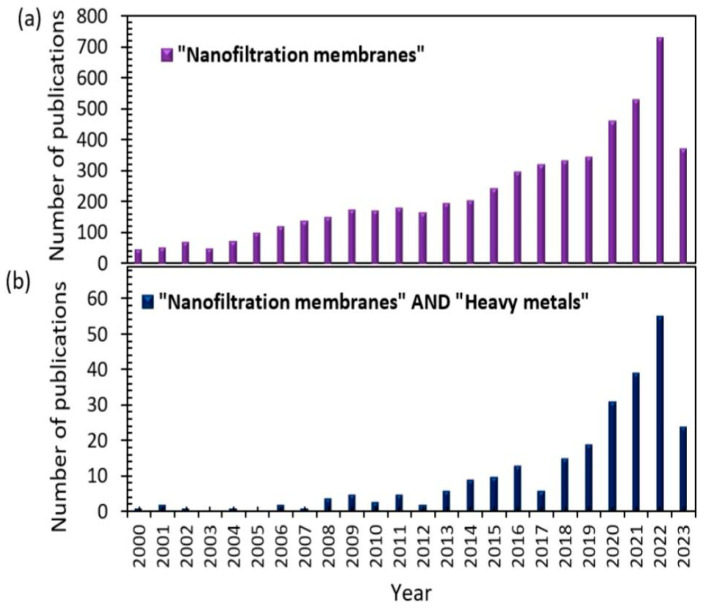

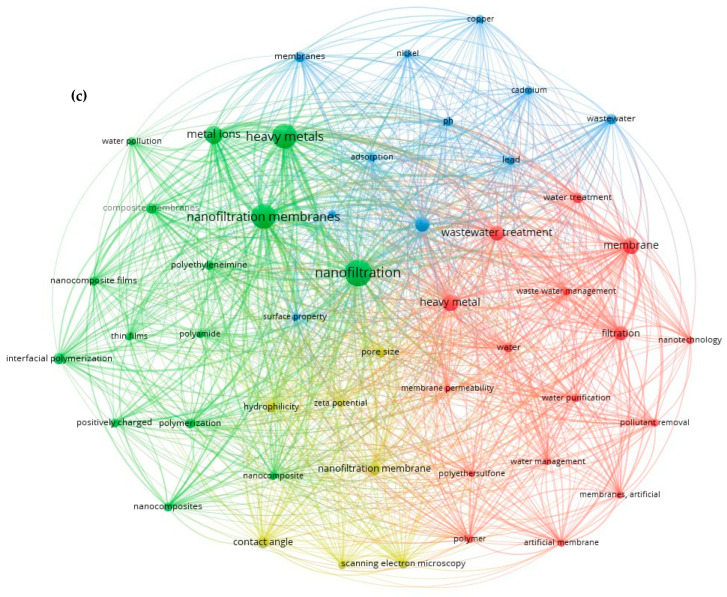
(**a**,**b**) Publications published since 2000 about (**a**) “Nanofiltration membranes” and (**b**) “Nanofiltration membranes” AND “heavy metals”. Source: Scopus 2023. (**c**) Network of keyword co-occurrences retrieved from Scopus database in 2023.

Nanofiltration (NF) is a new and effective method to remove heavy metals from impure water sources. This sophisticated filtering method uses semi-permeable membranes with pore sizes of 1–10 nm, permitting the selective removal of heavy metal ions from water while preserving vital minerals and nutrients. This makes the nanofiltration system an ideal method for eliminating heavy metals [7]. There is growing interest in the utilization of nanofiltration for the removal of heavy metals such as copper [8,9], cobalt [10], zinc [11], cadmium [12], mercury [13], lead [14], iron [15], chromium [16], nickel [17], manganese [18], antimony [19], and arsenic [20]. Figure 2 illustrates the increasing number of publications each year, especially recently, on heavy metal removal compared with the application of nanofiltration membranes in wastewater treatment and desalination.

The integration of nanomaterials improves the performance of nanofiltration membranes. Nanomaterials can be on the nanoscale, which makes membranes highly selective for heavy metals. However, further research is necessary to illustrate the performance of nanoparticles in nanofiltration. The modification of graphene oxide (GO) as a coating layer on polyamide (PA) resulted in enhanced membrane physicochemical characteristics and increased cobalt removal to 97% [21]. Carbon quantum dots (CQDs) were grafted onto polyethyleneimine (PEI) on a PA substrate via nanofiltration. CQDs enhance water permeability to 20.8 L m^−2^ h^−1^ bar^−1^, hydrophilicity, and antifouling characteristics for the removal of humic and phenyl acetate [22]. Employing Cu_2_O nanoparticles onto polyethersulfone (PES) via nanofiltration improved the water flux from 9.78 L m^−2^ h^−1^ to 36.78 L m^−2^ h^−1^, reducing the surface roughness and improving hydrophilicity. The heavy metal rejection was for Pb^2+^ (46%), Cu^2+^ (45%), and Cr^2+^ (49%). After the addition of nanoparticles, the rejection increased to 85.08%, 81%, and 79.38% for Pb^2+^, Cu^2+^, and Cr^2+^, respectively [14].

The recruitment of sodium dodecyl sulfate (SDS) as a surfactant with the plasticizer acetyltributylcitrate (ATBC) to polyethyleneimine (PEI) has a low cost, a high permeability of 9.7 L m^−2^ h^−1^ bar^−1^, and a rejection rate of >90% for MgCl_2_, Mn^2+^, Ni^2+^, Cd^2+^, and Cu^2+^ compared to bare PEI [18]. Table 1 shows the maximum contaminant limit and the possible hazards of these specific contaminants [5].

Based on the Scopus database, the analysis of keyword co-occurrence was conducted to build a network visualization, as shown in Figure 2c. Sixty keywords with the greatest link strength were selected, and the distance between the nodes closely reflected the terms. When the distance between nodes is closer, a strong association is identified between keywords such as nanofiltration membranes, heavy metals, composite membranes related to their characteristics, and different ions.

This review article provides a comprehensive overview of how nanoparticles markedly affect the nanofiltration membranes and assist their adsorption and removal of heavy metals by illustrating the mechanism of attachment of nanoparticles to NF membranes and by evaluating the performance of many nanofiltration membranes and comparing the different conditions applied in membrane fabrication and how that effect improves the performance of NF membranes in terms of flux, porosity, fouling, heavy metal, and salt rejection. Furthermore, it highlights the antifouling resistance of NF. Consequently, this review will contribute to the prevention of an increase in contaminant levels, which is important for its application in the environment and health.

## 2. Nanofiltration

**Figure 3:**
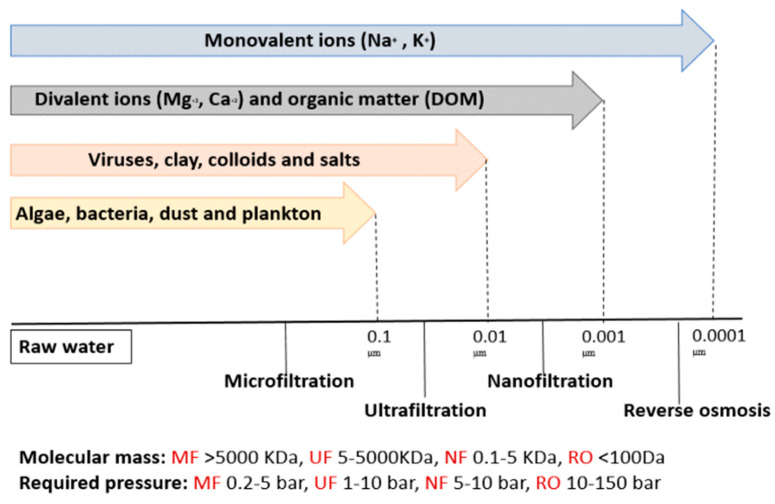
The representation of membrane types utilized in water treatment.

Nanofiltration is a type of membrane filtration that employs a semi-permeable membrane. Unlike other types of membrane filtration systems, nanofiltration membranes have pore sizes of less than 10 nm. It lies between ultrafiltration (UF) and reverse osmosis (RO) membranes, which allow the removal of divalent ions such as Mg^2+^, Ca^2+^, Pb^2+^, Co^2+^, Mn^2+^ and Zn^2+^, in addition to dissolved organic matter (DOM) (Figure 3) [23,24,25]. Pressure was forced through a semi-permeable membrane with pores smaller than those of heavy metal ions. The membrane allows water molecules to move while capturing heavy metal ions and other contaminants [26,27].

Fabrication techniques for nanofiltration (NF) involve two main processes: interfacial polymerization (IP) and phase inversion (PI). Notably, the aqueous and organic phases are the main components in the formation of the NF membranes through IP. Hence, some NF membranes may be used as initiators or catalysts. At the interface, polymerization occurs once the solutions are in contact. The monomers of the two solutions are divided, and the polymerization occurs through the film. This results in the formation of a thin film, and the thickness of the polymer film increases with time [28]. This process may include the reaction between a ligand with a vacant orbital and a metal ion with an extra lone pair to form a coordinate bond. Metal–ligand complexes in which the polymer film on the membrane surface are originally functionalized with ligands that possess a high affinity for heavy metal ions, such as carboxylic acid or amine groups. However, this model is uncommon in the fabrication of nanofiltration membranes [29]. The popular compounds used in the formation of metal-ligand complexes are metal–organic frameworks (MOF).

**Figure 4:**
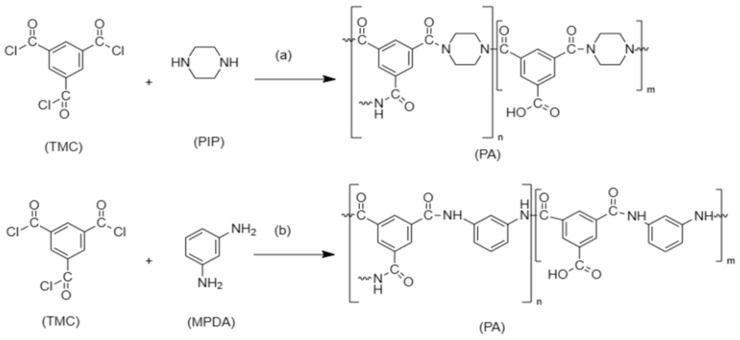
Interfacial polymerization to form TFC- PA. (**a**) reaction between PIP and TMC. Redrawn from [31]. (**b**) reaction between MPDA and TMC. Redrawn from [32].

Seah et al. [30] fabricated a nanofiltration membrane by reacting trimesoyl chloride (TMC) monomer ‘organic phase’ with piperazine (PIP) monomer ‘aqueous phase’. The IP then takes place to form the polyamide TFC, as demonstrated in Figure 4a. Another example is the interfacial polymerization between trimesoyl chloride (TMC) and m-phenylenediamine (MPDA) to form TFC polyamide, as shown in Figure 4b, which achieves great removal of heavy metals with the assistance of polyethersuflone (PES) as a support layer. The reaction took place between the chloride atom in (TMC) which started the reaction with the hydrogen atom in (MDPA) to form an amide bond [31].

**Figure 5:**
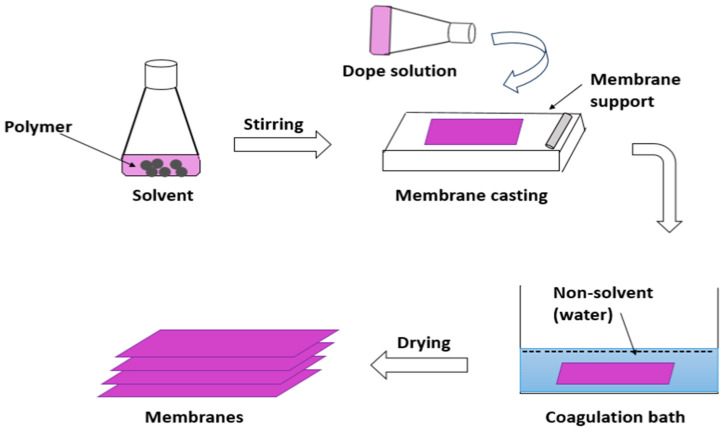
Immersion precipitation process in which the polymeric matrix dissolved with solvents to form membranes.

Another process is phase inversion by the introduction of non-solvent ‘water’ to form non-solvent-induced phase separation or, in some cases, is named ‘immersion precipitation’. This process is illustrated in Figure 5. where a polymer or polymer combination is dissolved in at least one solvent to form a dope solution. The dope solution is subsequently poured as a liquid layer onto a substrate, a glass plate, or a polymeric substrate [33]. It includes the creation of two phases by performing the exchange of a non-solvent from a coagulation bath, usually water, for the solvent from the polymer solution. A solution with a high polymer content is responsible for the production of the membrane matrix. The second phase includes only a very small amount of polymer [34]. After that, the membrane was dried in an oven or at room temperature and then ready to enter the nanofiltration system.

Fabrication of sheet membranes in NF utilizing chitosan/polyvinyl alcohol and montmorillonite clay was followed by a non-solvent-induced phase inversion technique and exhibited better performance in the removal of chromium [35]. The (NF) membrane was fabricated with the help of a Spinneret incorporating a dry-jet wet. The introduction of polybenzimidazole and polyethersulfone/polyvinylpyrrolidone (PBI-PES/PVP) dopes was introduced through a phase inversion process [36]. Positively charged, highly permeable nanofiltration membranes were fabricated by the phase inversion method using the poly(acid–base) complexation effect for heavy metal elimination [37].

The membrane structure is composed of three layers (1) a nonwoven polymeric support ‘’polyethylene terephthalate’’, (2) a microporous polymeric support ‘’polysulfone’’, and (3) a thin separation layer consisting of cross-linked ‘’polyamide’’ [38]. Polyaniline nanoparticles were added to polysulfone/chitosan by phase inversion to form a nanofiltration membrane. The water flux recorded was 8.04 L m^−2^ h^−1^. The rejection of copper was >86% [39]. The engagement of polyethersulfone (PES) by 0.5 wt% to graphene oxide (GO) via phase inversion. This resulted in the formation of sulfonated graphene oxide (sGO-0.5) nanoparticles, which resulted in a dense membrane and formed a finger-like structure with mass growth. It exhibited the best performance in comparison with bare polyethersulfone (PES) and graphene oxide (GO). The rejection of heavy metals was the best in sulfonated graphene oxide (sGO-0.5), in the order of Cr > Cd > Cu > Ni. Additionally, sGO-0.5 exhibited the best performance in terms of resistance to fouling [40].

Ongoing research aims to introduce new fabrication methods for the removal of heavy metals by nanofiltration. Among various methods to remove contaminants from discharged land streams, nanofiltration is favoured over other processes because of the innovation of nanoparticles. Nanoparticles produce membranes with high simplicity, manufacturing scalability, and energy efficiency [41]. Nanofillers are forms of nanoparticles that contribute to membrane filtration by enhancing the thermal, chemical, and mechanical properties of membranes as well as membrane separation characteristics. The conduction of nanofillers to the membrane surface facilitates the compatibility between the fillers and the polymeric matrices, which mainly includes the surface modification of nanomaterials with reactive moieties [42].

To estimate the performance of the nanomembrane, several parameters such as water flux, porosity, hydraulic resistance, salt rejection, and surface roughness can be obtained. These parameters are key factors in the maintenance of selectivity, permeability, and fouling resistance at optimum levels [43].

Water flux is the flow of water, in which the volume of permeate per unit area per unit time. The relationship between the water flux and pressure could determine water permeability. However, both the operating pressure and feed salt concentration have a significant impact on salt transport during the feed water filtration process [44,45]. The nanofiltration membrane is a dense membrane. The capacity to retain water is termed porosity (*η*), which is characterized by the volume of the vacant space and is usually in the range of ~10–35%. Equation (1) labels the relationship between (*V_voids_*), the volume of void space as water, and (*V_total_*), the total volume of the system under study. This can be outlined in the form of a percentage, as given in Equation (2). Porosity can be calculated from the densities of polymers *ρ_f_* (g cm^−3^) and blended membrane *ρ_p_* (g cm^−3^) as given in Equation (3) [46].

(1) $$\eta =\frac{V_{voids}}{V_{water}}$$

(2) $$\eta =\frac{V_{voids}}{V_{water}}\times 100$$

(3) $$\%\eta =\left(1-\frac{\rho_{f}}{\rho_{p}}\right)\times 100$$

The density of the blended membrane (*ρ_p_*) (g cm^−3^) is the combination of all polymers where (*p*_1_) is the density of the first polymer, and (*w*_1_) is the polymer’s weight. (*p*_2_) is the density of the second polymer, and (*w*_2_) is the polymer’s weight, which is represented by Equation (4). The density of polymers (*ρ_f_*) (g cm^−3^) is given by Equation (5) where L is the fiber length (m), d_i_ is the inner diameter, and *d_o_* is the outer diameter of the membrane (m) [46].

(4) $$\rho_{p}=\rho_{1}w_{1}+\rho_{2}w_{2}$$

(5) $$\rho_{f}=\frac{4\;w}{\pi \;L\;\left(d_{o}^{2}-d_{o}^{2}\right)}$$

The difficulty of a fluid moving through a pipe or channel is known as hydraulic resistance. The hydraulic resistance (*R_m_*) of the transmembrane in water treatment refers to the resistance to water flow through a membrane. This is a crucial factor that influences how well membrane filtration systems are used for water treatment. Hydraulic resistance of the membrane is calculated from *TMP* (transmembrane membrane pressure) ‘psi’ is gauged over time by water flux by Equation (6) [46].

(6) $$R_{m}=\frac{TMP}{J_{w}}$$

“Salt rejection” is the opposite of “salt passage” and refers to the movement of salt through the membrane barrier layer. Rejection (*R*) can be calculated using the salt concentration in the permeant flow (*C_p_*) and salt concentration in the feed flow (*C_f_*). Rejection is always calculated in percentage as given in Equation (7) [47].

(7) $$\%R=\left(1-\frac{C_{p}}{C_{f}}\right)\times 100$$

Surface roughness (*R_a_*) is a measure of the texture of a surface. The distinction between membrane surface topography and a hypothetical, atomically flat surface can used to describe the roughness of membranes. It has many characteristics, such as the pore-size distribution and molecular weight. Surface roughness is calculated from Equation (8). In which (*N*) is the total number of points at a given area, (z) is the height at point ‘n’, and (z^−^) is the (height) of the center plane [48].

(8) $$R_{a}=\sum_{n=1\;}^{\;N}\frac{\left|z-z^{-}\right|}{N}$$

Fillers with particle sizes between 1 and 100 nm are frequently referred to as nanofillers [49]. Regarding membrane separation, nanofillers can be made of (1) carbon-based materials such as ‘graphene oxide (GO), carbon nanotubes (CNTs), and activated carbon’, (2) metal oxides (inorganic fillers) such as ‘ZnO, Al_2_O_3_, SiO_2_, and TiO_2_, (3) metal–organic frameworks (MOFs) such as ‘MIL-101 and UiO-66′ [50,51] or (4) polymers such as ‘hyperbranched polymers, polyphenol derivatives, and polymeric nanofiber’ [52]. (5) Clay nanofillers such as montmorillonite, kaolinite, laponite and hectorite. Three distinct groups can be used to sort nanofillers in the form of nanoparticles of three different sizes: one nanoscale (nanoplatelets), two nanoscales (nanofibers), and three nanoscales (nanoparticulates) [18].

In contrast, nanofillers are introduced into the membrane matrix to achieve better performance and low membrane fouling, which is the inappropriate deposition and build-up of particulate matter, microorganisms, colloids, and solutes over the surface of the membrane [53]. In addition, an increase in antifouling properties prevents membrane fouling. Some examples of nanofiltration membrane fabrication with the assistance of nanofillers are as follows:

### 2.1. In-Situ Polymerization

Nanofillers were introduced into the membrane matrix via polymerization with monomers to form the nanocomposite membranes. This is a superior method for controlling membrane thickness. According to Tong et al. [54], the utilization of silica as a filler in the salt rejection of NaCl was 98.5%. In situ polymerization of tetramethyl orthosilicate in the polyamide layer. The resulting nanofiltration membrane recorded a water flux of 72.77 L m^−2^ h^−1^. TFNs were prepared by in situ interfacial polymerization between polydopamine and piperazine nanoparticles. The NF membrane exhibited high water permeability, salt selectivity, and water flux of 59.1 ± 3.3 L m^−2^ h^−1^ [55].

### 2.2. Layer-by-Layer Assembly

This includes the aggregation of nanoparticles on polymeric materials to form nanocomposites with multilayered forms, which may be organic and inorganic nanocomposites [56]. The UiO-66-NH_2_ nanofiller was added to a polyamide (PA) thin film to form a UiO-66-NH_2_/PA thin-film composite (TFC), which was used in the nanofiltration technique for lead removal [57]. Another fabrication of nanocomposites was made by Li et al. [8] by the incorporation of polyamide (PA) via a surface grafting method using poly(amidoamine) dendrimer (PAMAM) for the removal of Cu^2+^, Ni^2+^, and Pb^2+^ by nanofiltration. Demonstration of UiO-66-(COOH)_2_ nanofiller onto reduced graphene (rGO) to form a nanofiltration thin film composite that recorded a rejection of Cu^2+^ (96.5–83.1%) and Cd^2+^ (92.6–80.4%) and pure flux of (20.0 ± 2.5) L m^−2^ h^−1^ bar^−1^ [58]. Nanofiltration membranes can be fabricated using GO nanofiller sheets, which are added to polyacrylic acid and polyethersulfone (PES) to remove heavy metals [59].

### 2.3. Blending

Nanofillers are blended with a polymeric mixture, solvents, or additives to form a doping solution before casting to form a nanocomposite membrane. The blending depends on the concentration of the polymers and the type of nanofiller used. This enhances the properties of nanocomposite membranes by increasing their mechanical strength, diminishing surface fouling, and escalating heavy metal removal. This method is used to produce ceramic/binder, metal/binder, and polymer/filler composite powder [60].

Preparation of nickel-bentonite nanoparticles (NBNP) with polyethersulfone (PES) to upgrade a nanofiltration membrane with outstanding antifouling properties for the removal of Fe^2+^, Pb^2+^, Zn^2+^, and Cu^2+^. The recorded total porosity was 71.6–83.8%, and the water flux of 44.11–78.34 kg m^−2^ h^−1^ reached 98.2% [11]. Fe_3_O_4_ nanoparticles were applied to SiO_2_ in the formation of nanofiltration membranes with a flux of 70.6 L m^–2^ h^−1^ to remove Pb^2+^, Cd^2+^, and Cu^2+^ [12]. Chitosan (CS) was blended with multi-walled carbon nanotubes (MWCNTs) to form a nanofiltration membrane with a registered flux of (~9.41 kg m^−2^ h^−1^) for the removal of Cu^2+^ and Ni^2+^ [17].

### 2.4. Coating

Nanofillers are deposited onto the nanofiltration membrane during coating to create a coated thin film. This may be because the dip coating notably resembles phase inversion, in which the nanofiller is dipped into a solution, and then the unwanted extra solution is removed by drying to provide mechanical and corrosion resistance. Spin coating is achieved by electrospinning, where the nanofiller is added to the membrane surface and spun at a high speed in the last step. Electrospinning begins by pumping a polymer solution through a narrow capillary in an elevated state of a strong electric field (on the order of kV cm^−1^) between the capillary and a grounded collector at a low flow rate (on the order of mL h^−1^). A liquid droplet forms near the capillary tip, which is deformed by an electric field into a (Taylor cone) [61].

The aniline oligomer was coated with tetrathioterephthalate and contrasted to form a nanofiltration membrane with a high tendency to remove Pb^2+^ and Cu^2+^ with an increase in the flux percentage of 175.06% and high antifouling resistance [62]. Multiwalled carbon nanotubes (MWCNTs) were functionalized with –COOH and reacted with polyethylenimine (PEI). Polyacrylonitrile (PAN) was used as a nanofiber via electrospinning to improve the reaction. The recorded flux was 145.8 L m^−2^ h^−1^ to remove Pb^2+^ and Cu^2+^ with efficiency > 84% [63].

## 3. Mechanism of Removal of Heavy Metals by NF

Nanofiltration membranes use membrane micropores to filter and employ the selective permeability of membranes to separate particulates from wastewater. The nanofiltration membrane acted as a barrier in the separating feed solution. These barriers control the passage of species. Membrane pore size has an effective selectivity for heavy metals. The smaller the membrane pore size, the higher the separation of heavy metals. Steric hindrance is responsible for the rejection of ions with diameters larger than the pore size of the membrane [64]. Size exclusion and the Donnan effect are considered important methods for measuring membrane efficiency during separation [65]. Size exclusion occurs when the ions of feed solution at different sizes are introduced onto the nanofiltration membrane, and the membrane starts to select specific ions to pass through it [66]. The Donnan effect is another mechanism of separation with the assistance of a semipermeable membrane, in which selective ions assemble on one side of the barrier and undesired ions are on the other side [67]. In nanofiltration techniques, the Donnan effect is widely used and effective for ion separation [68].

Shao et al. [69] formed a PEI/GO (polyethyleneimine/graphene oxide) thin film nanocomposite and used size exclusion and the Donnan effect to measure the selectivity of ions. PEI/GO. The membrane exhibited a positive surface charge and sharp repulsion with monovalent and divalent ions. However, the membrane possesses a full-fed attraction to negatively charged ions. Rejection mechanisms include (1) the formation of hydrogen bonds between water and membrane molecules in which heavy metals are transported. (2) Electrostatic repulsion is another rejection mechanism towing to the difference in the dielectric constant between the membrane and solution. (3) The charged capillary mechanism is the electric double layer in which ions of the same charge of the membrane are attracted while different charges are rejected. (4) Diffusion mechanism by the desolvation of the solute and solvent, diffusion of the solvent, and diffusion through the membrane [70].

## 4. Nanoembedded Membranes

**Figure 6:**
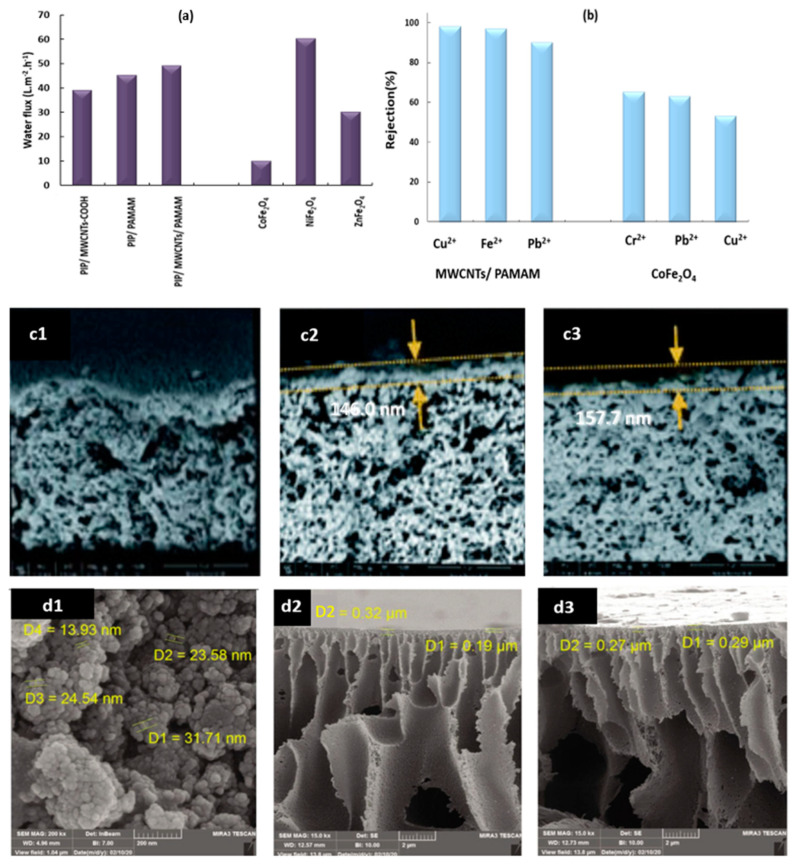
(**a**) Relationship between pure water flux and the various forms of nano-embedded membranes, (**b**) Rejection of heavy metals by nano-embedded membranes, (**c1**) PIP/PAMAM, (**c2**) PIP/MWCNTs, (**c3**) MWCNTs/PAMAM, (**d1**) CoFe_2_O_4_, (**d2**) PES and (**d3**) CoFe_2_O_4_/PES [15,16].

It is a type of nanofiltration membrane in which nanoparticles in the form of nanofillers are embedded in the polymeric matrix to enhance surface roughness, selectivity, and porosity [71]. The use of these small nanosized particles in membranes has several advantages and disadvantages. A superior connection between the two phases of the membrane results in enhanced selectivity, permeability, mechanical stability, hydrophilicity, and reduced fouling, among other benefits. Alumina, TiO_2_, Attapulgite (APT), zeolite, zinc oxide, and silica are among the most popular nanoparticles used in polymeric and ceramic membranes [19,72]. Polyamidoamine (PAMAM) was incorporated into acidified multi-walled carbon nanotubes (MWCNTs), and the resulting membrane was embedded in polyamide (PA) by interfacial polymerization. Interfacial polymerization of PA occurs between trimesoyl chloride (TMC) and piperazine (PIP). The water flux was dramatically enhanced after the addition of MWCNTs/PAMAM, reaching 48.7 L m^−2^ h^−1^, as shown in Figure 6a. Figure 6b reveals that the heavy metal rejection (%) was the highest with the MWCNTs/PAMAM membrane for Cu^2+^, Fe^2+^, and Pb^2+^. This indicates the capacity of the nano-embedded membrane to reject multiple heavy metals at a constant concentration of 300 mg L^−1^ and acidic pH. The mass of the membrane increased because of nanoparticle embedding, as shown in Figure 6c. Figure 6(c1) shows the untreated membrane, and the mass was slightly increased by the addition of MWCNTs (Figure 6(c2)) and greatly increased by embedded PAMAM in Figure 6(c3) [15].

Ferrite nanoparticles were implemented to form nanofiltration-embedded membranes in the form of CoFe_2_O_4,_ NiFe_2_O_4_, and ZnFe_2_O_4_ on polyethersulfone (PES). The addition of ferrite nanoparticles reduced the membrane pore size to 65.3% and reduced the pore size to 1.3 nm, which produced a high water flux, as shown in Figure 6a. The rejection rate of Cr^2+^ (78%), Pb^2+^ (72%), and Cu^2+^ (75%), as measured by CoFe_2_O_4_ in Figure 6b exhibited a strong performance of the membrane. Substantial evidence of the effect of nanoparticles on improving the membrane is shown in Figure 6d. The membrane diameter is increased in Figure 6(d1) in comparison with the bare membrane in Figure 6(d2) [16].

## 5. Nanocomposites

Nanocomposite membranes, prepared by mixing nanofillers with polymeric membrane matrices, can enhance filtration. Nanocomposite membranes usually demonstrate improved antifouling properties, which reduces the roughness of the membrane [73]. Numerous possibilities for nanocomposite membranes involve desalination and elimination of multiple impurities during water treatment procedures. Several types of nanoparticles, carbon-based materials, and polymers have been used for this purpose [74]. The incorporation of nanocomposites into nanofiltration membranes has a significant impact on the removal of heavy metals. The addition of combined nanoparticles leads to a sharp improvement in water permeability, selectivity, and antibacterial properties [75]. Nanocomposites can also be formed from other nanoparticles, polymers, and biopolymers. Nanocomposites may contain the same types of nanofillers, such as metal oxide and carbon-based nanocomposites. They have high adsorption capacities for heavy metal removal. Polymer-based nanocomposites can improve membrane performance, metal–organic framework nanocomposites with high surface areas, microporous zeolite nanocomposites with a high tendency for heavy metals removal, bio-based nanocomposites, and biopolymers (cellulose and alginates). Table 2 describes the many nanocomposite materials and polymeric matrices used, the method of fabrication of nanofiltration membranes, and the membrane efficiency in a simplified structure for the removal of heavy metals. This Table focuses on the membrane performance measurements in the form of water flux, which is the water flow rate at a given time. In addition, a brief description of the impact of the nanocomposite on nanofiltration membranes is provided [76].

**Figure 7:**
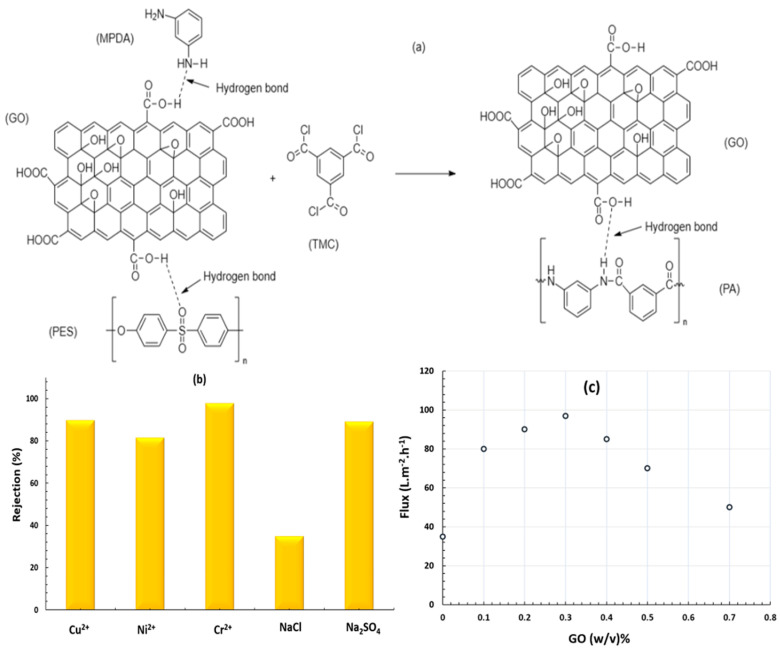
(**a**) Cross-liking reaction can cause the formation of PA/GO thin film nanocomposite, (**b**) representation of the rejection of heavy metals and salts by PA/GO TFN, (**c**) relationship between water flux and the concentration of GO. Redrawn and data derived from [91].

Fouladi M et al. [91] fabricated thin film nanocomposite (TFN) by modification of graphene oxide (GO) onto polyamide (PA), which was obtained from interfacial polymerization between trimesoylchloride (TMC), polyethersulfone (PES), and m-phenylenediamine (MPDA), as illustrated in Figure 7a. The resulting PA/GO membrane can be formed by cross-linking via the formation of hydrogen bonds. Salt and heavy metal rejection are given in Figure 7b, which has been recorded at a constant pH = 7 and a pressure of 15 bar, indicating the negatively charged and high hydrophilicity of the membrane. This also reflected the high adsorption power of the membrane after the incorporation of GO nanoparticles. The order of rejection was Cr^2+^ (97.5%) > Cu^2+^ (89.57%) > Ni^2+^ (83.37%) with strong salt rejection of divalent ions. The experiment tested a high-water flux when the addition of GO nanoparticles jumped from 89.7 L m^−2^ h^−1^ for bare PA to 97.98 L m^−2^ h^−1^ for PA/GO at 15 bar. The more increments in the concentration of GO nanoparticles, the higher the flux recorded when the loading is 0.3 (*w*/*v*)% of GO nanoparticles, as illustrated in Figure 7c. From the previous paragraph, the types of nanofillers have a significant impact on the nanocomposite for nanofiltration membrane fabrication. Additionally, the effectiveness of the nanofiltration membrane in removing heavy metals is affected by some operating factors [92].

### 5.1. pH

**Figure 8:**
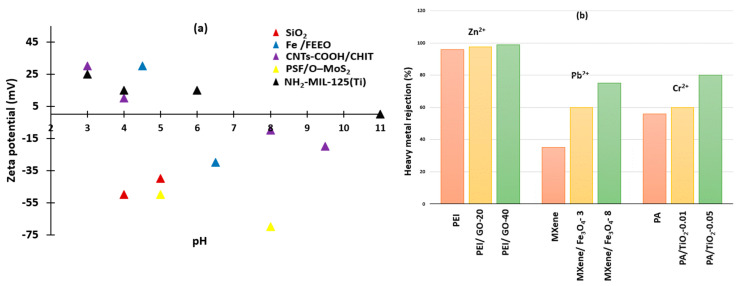
(**a**) Representation of the compaction of pH at the nanocomposite behavior of the nanofiltration membrane. Data were retrieved from [82,87,92,93,99]. (**b**) Schematic representation of the impaction of nanocomposite materials at different concentrations compared with untreated nanofiltration membrane in the rejection rate of Zn^2+^, Cr^2+^, and Pb^2+^. Data are retrieved and drawn from [69,97,98].

It is the hydrogen potential that determines the acidity or basicity of a substance. However, nanofiltration membranes determine the surface charge of the membrane. At low pH, the surface becomes saturated with positive hydronium ions. Repulsion force occurs between the positively charged metal ions and (H_3_O^−^). The repulsion force highly reduced the membrane’s ability to remove heavy metals and resulted in increasing the membrane fouling. On the other hand, at high pH, the surface becomes more negative and diminishes the repulsion force, then increases the adsorption power of heavy metals between the surface of the membrane and heavy metals. Therefore, it causes a reduction in membrane fouling properties. Zeta potential is a function of pH, which is reflected in membrane wettability [82,87,92,93]. Figure 8a ensures the main idea of the regulation of pH on the surface charge by comparison between various types of nanocomposite materials. Zeta potential can explain the behavior of (Fe/FEEO), (CNTs-COOH/CHIT), and (NH_2_-MIL-125(Ti)) at low pH, they possess a positive surface charge, and at high pH, they have a negative surface charge. However, that effect cannot be applied to (SiO_2_) and (PSF/O-MoS_2_) because they recorded only a negative surface charge at low, moderate, and high pH.

### 5.2. Temperature

The increment of temperature but in a moderate manner can significantly increase the elimination of heavy metals by nanocomposites [94]. Increasing the temperature affects the cross-linking between the composite material and the heavy metals [95]. Extra high temperatures ≥80 °C can cause membrane degradation. At very high temperatures, the solvents made the membrane evaporate, causing a loss of membrane weight. According to Alhoshan et al. [96], the fabrication of nanofiltration membranes is made by the incorporation of polyaniline (PANi) nanoparticles into polyphenylsulfone (PPSF) to form a nanocomposite for the extraction of Cd^2+^ and Pb^2+^. At temperatures between 200 °C and 500 °C, the membrane lost 10% of its weight, while at temperatures of 500 °C and 700 °C, the membrane converted to ash.

### 5.3. Concentration of Feed Solution

Increasing the concentration of the feed solution increases the ion concentration, which is reflected in the increase in heavy metal concentration. Additionally, the use of nanoparticles at different concentrations can prevent this type of interruption. The rejection of metal ions, as depicted in Figure 8b, can be achieved by increasing the concentration of the nanocomposite materials, which helps in the membrane separation process [69,97,98]. For Zn^2+^ rejection, graphene oxide (GO) nanoparticles were incorporated into polyethyleneimine (PEI) to form a thin film nanocomposite (TFN). The recorded flux was 70.3 L m^−2^ h^−1^. At different concentrations of GO (20 ppm and 40 ppm), the recorded zinc rejection was the highest for GO-40 ppm and GO-20 ppm, and bare PEI recorded the lowest rejection of zinc. This means that an increase in the nanoparticle concentration indicates a better separation performance of heavy metals [90]. Ferric oxide (Fe_3_O_4_) nanoparticles were added to MXene sheets at different amounts of 3 mg and 8 mg to eliminate Cr^2+^, which resulted in the highest removal of Cr^2+^ by MXene/Fe_3_O_4_-8; then, MXene/Fe_3_O_4_-3 and untreated MXene recorded the least removal ability of chromium [97]. TiO_2_ nanoparticles were added to polyamide (PA) in different concentrations of 0.01 (*w*/*v*)% and 0.05 (*w*/*v*)%. In contrast, the water fluxes were 25, 39, and 41 L m^−2^ h^−1^ for the untreated (PA), (PA/TiO_2_-0.01), and (PA/TiO_2_-0.05), respectively. This means that high rejection power in the removal of Pb^2+^ was achieved by increasing the concentration of nanoparticles (PA/TiO_2_-0.05) > (PA/TiO_2_-0.01) > (PA) [98].

### 5.4. Pressure

The increment in pressure not only increases the adsorption power of heavy metals but also increases the selectivity and water permeability of the membrane [99]. Considering that the nanofiltration membrane is a pressure-based method, it is allowable to pass the ions at pressures between 5 and 20 bar. Operation pressures <5 or more than 20 are not permissible for nanofiltration.

## 6. Mitigation Fouling of NF Membranes

**Figure 9:**
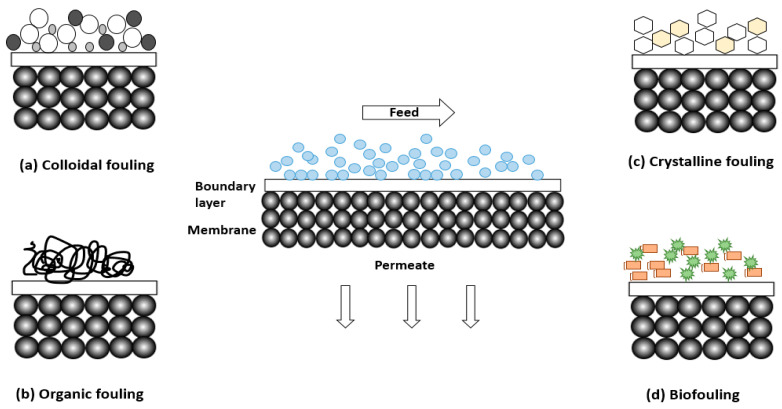
Fouling membrane types.

When water flows in the form of a feed solution to enter the membrane surface, some ions attach to the surface. This reduces the effectiveness of the membrane and is termed ‘membrane fouling’. Membrane fouling involves the aggregation of pollutants on the membrane surface. Particle size is a consequence of the fouling mechanism of the membrane filtration system. If the size of the foulant is smaller than the pore size (i.e., solutes) or is equivalent to the diameter of the membrane’s pores (i.e., colloids), significant adsorption at the internal pore surface and pore-clogging can occur. If the pores are significantly larger than the holes in the membrane, the foulant tends to develop a cake layer on the membrane surface. Foulants affect membrane performance and might accumulate in basins on the surface of membranes that have a rough texture [100]. All pressure-driven membrane separation systems face the major issue of membrane fouling, which shortens the lifetime of the membrane and lowers the flow [101]. Figure 9 shows several types of membrane fouling. Deposits of crystalline fouling (such as mineral precipitates), colloidal fouling (such as clay and flocs), organic fouling (such as humics, oils, and polyelectrolytes), and biofouling (such as bacteria and fungi) are different types of fouling [102]. Antifouling membranes have been developed to prevent membrane fouling. Antifouling membranes are one of the most vibrant and essential methods to treat water [103]. Antifouling membrane technology illustrates how they can reduce costs by replacing pre-treatment techniques, consuming less energy, decreasing cleaning frequency, and extending membrane lifetimes [104]. To be equipped with membranes, fouling can be avoided by plenty of techniques such as pre-treatment of feed, membrane, surface modification by antifouling agents, membrane selection, and operating conditions optimization.

### 6.1. Pre-Treatment of Feed Solution

Pre-treatment of feed solution aims to endure the membrane by increasing its permeability and stability and decreasing the fouling characteristics. Pre-treatments possess adsorption, coagulation, and flocculation [105]. Adsorption of heavy metals can be performed by relative adsorbents such as carbon nanotubes (CNTs), graphene, activated carbon (AC), chitosan (CS), Fe_3_O_4_, clay, and UiO-66. However, some drawbacks in adsorption are related to surface area, retention time, and the stability of adsorbents [106]. Flocculation and coagulation usually occur together; coagulants such as aluminum–sulfate and ferric–chloride bind to the heavy metal to neutralize their charges, and flocculants such as polyferric–sulfate and polyacrylamide bind the particles together. However, the processes required a long time and several steps, in addition to the cost effect of including more than one chemical [107].

### 6.2. Surface Modification

The degree of cross-linking among materials is a strong indication of how the interconnection takes place. Antifouling agents can form two types of surface modification: 2D modification, which includes surface coating and surface grafting, and 3D modification, which includes physical blending and surface segregation [99].

**Figure 10:**
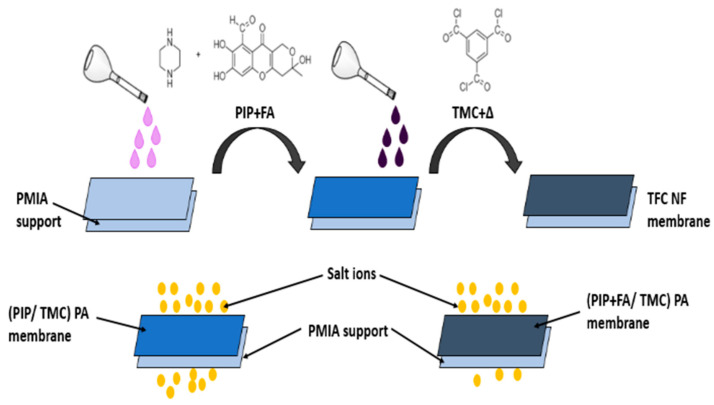
Mitigation of fouling by coating FA on PA/PMIA nanofiltration membrane.

Coating a membrane with an antifouling material is a method of preventing membrane fouling with the presence of heavy metals. These coatings provide a thick hydration layer that prevents foulants from adhering to the membrane surface. The coating intends to prolong the flux recovery following cleaning. Anti-fouling coatings add hydrogen bond acceptors while avoiding hydrogen bond donors and providing the surface with an overall neutral electrical charge. This increases the surface hydrophilicity but not significantly [108]. Figure 10 describes the interfacial polymerization between piperazine (PIP) and trimesoyl chloride (TMC) to form polyamide (PA) that is poured onto poly (*m*-phenylene isophthalamide) (PMIA) and fulvic acid (FA) as an additive via nanofiltration, which exhibited a strong antifouling property and flux of 22.3 L m^−2^ h^−1^ bar^−1^ and salt rejection reached 97.8% [109].

**Figure 11:**
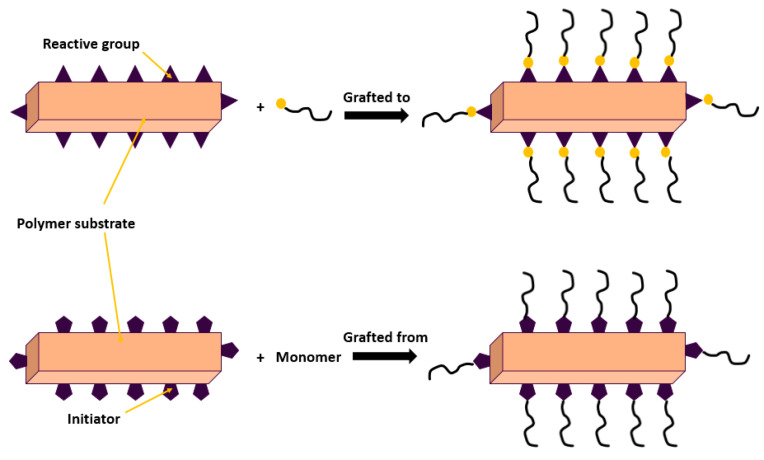
Mitigation of fouling by “grafting to” and “grafting from” techniques.

Surface modification tuned by grafting is the attachment of polymer chains with their functional groups to a membrane surface. Reduced foulant absorption will lead to good membrane antifouling properties as a result [110]. The so-called graft-to techniques entail grafting reactive terminal groups onto the prefabricated polymer chains. As an alternative, graft-from techniques, which involve growing a polymer in situ from the surface using an initiating group that has been surface-adsorbed, typically result in thicker polymer layers, as illustrated in Figure 11 [111]. Grafting increases the surface’s hydrophilicity. Numerous methods for the grafting of polymers involve electron beam, microwave grafting, plasma, and UV photographing.

One of the most practical methods for industrial scale production without any pre- or post-treatment is blending modification [112]. By incorporating additives such as (metal nanoparticles, carbon nanomaterials, and graphene) into a casting solution and encapsulating them in a polymer matrix, a series of nanocomposite membranes with anti-fouling surfaces have been created [113]. For thin-film nanocomposite (TFN), despite the interfacial polymerization step, the additive and monomer are blended [114].

Segmentation may occur during the fabrication of the nanofiltration membrane via phase inversion. Hydrophilic segments of an amphiphilic copolymer are segregated into hydrophobic and hydrophilic membrane surfaces; hence, the hydrophobic segment is segmented into the polymer matrix. Block copolymers, comb copolymers, and branched copolymers can be blocked by amphiphilic copolymers [115]. The driving force and relative diffusion rate of the solvent and non-solvent during the phase change can be reduced by the hydrophobic segment of the membrane. Separation causes the cast solution to take longer to complete the process of membrane construction, which ultimately results in the establishment of a sponge-like structure [116].

### 6.3. Membrane Selection

The selectivity of the membrane material and pore size had a sharp impact on heavy metal evacuation. Foulants form a cake layer on the membrane surface, which can be reduced by cleaning to eliminate physical fouling caused by backlash and air bubbles. Chemically, surfactants, detergents, and disinfection are used [117]. Fouling cleaning is based on the operational conditions of foulant concentration and pH [118]. Currently, research is ongoing on imparting separation techniques by membrane filtration pre-treatment. Some operational conditions, such as pH, temperature, pressure, and concentration, are essential factors in the mitigation of nanofiltration membrane fouling and the description of how the membrane surface can be modified, as previously discussed. Table 3 summarizes the nanofiltration process for reducing the fouling effect by various modification techniques in the presence of nanoparticles, a common advantage after the contribution of antifouling agents and flux as performance parameters.

Heavy metals exhibit fouling in two ways: (1) precipitates of heavy metals directly impact the fouling of the sludge, which is a biological precipitate in lakes and ponds, and (2) heavy metals affect the metabolism of organisms and interrupt the membrane causing fouling, which consists of sludge flocs, colloids, biopolymer aggregations, extracellular polymeric substances, dissolved organic matters, soluble microbial products, and inorganic substrates [125,126]. Nanocomposites act as antifouling agents to reduce the fouling effect of membranes [127]. Sulfonation of graphene oxide (GO) by polyethersulfone (PES) to a sulfonated graphene oxide (sGO) nanoembedded membrane through elimination of Ni, Cr, Cu, and Cd. The sequenced nanofiltration membrane is improved by the addition of bovine serum albumin to eliminate the fouling properties of the membrane [40].

**Figure 12:**
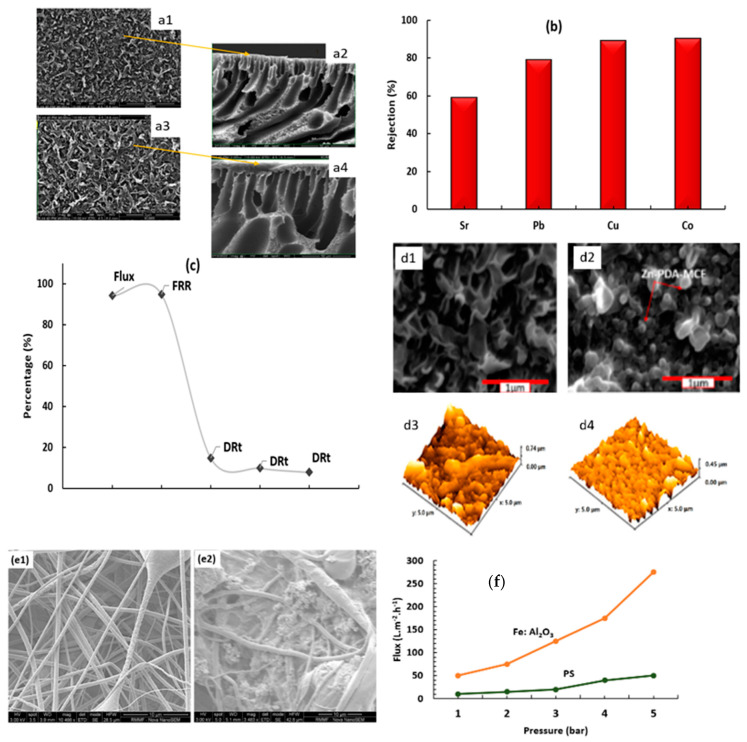
(**a1**) Upper layer of PS/PAMAM, (**a2**) PS/PAMAM membrane, (**a3**) upper layer of PS/PAMAM/TiO_2_, which is more porous than PS/PAMAM, (**a4**) PS/PAMAM/TiO_2_ membrane which high thickness compared to PS/PAMAM with finger-like structure. (**b**) Rejection of heavy metals by PS/PAMAM/TiO_2_, (**c**) relation between the addition of Zn-PDA-MCF-5 nanoparticle on improving the total flux, flux recovery ratio (FRR) and reducing the decline flux ratio (DRt), (**d1**) PA, (**d2**) PA/Zn-PDA-MCF-5 TFN with leaf-like structure, (**d3**) highly surface roughness of PA substrate and (**d4**) PA/Zn-PDA-MCF-5 TFN with low surface roughness. (**e1**) Pristine polyethersuflone/GO-ZnO-iron oxide nanoparticles, (**e2**) pristine polyethersuflone/GO-ZnO-iron oxide nanoparticles after treatment with BSA. (**f**) Relation between the total flux and pressure, which discussed the intensive increase in water flux when added Fe/Al_2_O_3_ compared to bare PS. Adapted and redrawn from [20,76,127,129].

Ranjbaran et al. [76] studied a nanofiltration membrane to determine the effect of the inclusion of nanoparticles on the reduction of nanofiltration membrane fouling and the improvement of resistance to heavy metals. TiO_2_ nanoparticles were incorporated with polysulfone (PS) via interfacial polymerization in the presence of the dendrimer ‘poly(amidoamine) (PAMAM)’. The addition of titanium dioxide nanoparticles resulted in an increase in the thickness and porosity of the membrane, as shown in Figure 12a. The water flux climbed from 68.36 L m^−2^ h^−1^ to 125.36 L m^−2^ h^−1^ by the addition of 0.05 wt% TiO_2_ in comparison with untreated PS/PAMAM, which caused the massive resistance of deposits on the membrane surface from water permeate. Figure 12(a1) shows the upper layer of the PS/PAMAM, which retained a low porosity in comparison with the upper layer of PS/PAMAM/TiO_2_ in Figure 12(a3). In addition to the high thickness of PS/PAMAM/TiO_2_ shown in Figure 12(a2,a4). Figure 12b shows high heavy metal rejection for Co (89.65%), Cu (86.56%), Pb (72.64%), and Sr (57.36%). The study explained the high resistance of PS/PAMAM after the addition of 0.05 wt% TiO_2_. Bovine serum albumin (BSA) increases pollutant resistance when added to PAMAM. Bovine serum albumin (BSA) was used as a protein fouling by embedded Zn-PDA-MCF-5 (mesoporous cellular foam) to form thin film nanocomposites with polyamide (PA) with a loading of 0.25 wt% via interfacial polymerization. Examination of the water flux (6.40 L m^−2^ h^−1^). By measuring the pure water flux recovery ratio (FRR) after the addition of nanoparticles, the water flux increased to 94.2%, and the flux decline ratio (DRt) was reduced to 16.14%, as shown in Figure 12c. The increment of water flux is referred to as the smoother and less rough, as illustrated in Figure 12(d4) membrane after the integration of Zn-PDA-MCF-5 on the reverse side in Figure 12(d3). The formation of a leaf-like structure in Figure 12(d2) on the nanofiltration membrane surface ensures a substantial idea of the effectiveness of membrane antifouling on the membrane performance after the addition of nanoparticles compared to the membrane in Figure 12(d1) [127]. Siddique et al. [20] incorporated graphene oxide/zinc oxide (GO/ZnO) and GO/ZnO/iron oxide nanoparticles into pristine polysulfone by electrospinning to eliminate Arsenic via nanofiltration. The surface area and porosity of the thin film nanocomposite cumulate a growth in surface area of 11.4% and diminish the pore size to 69% (Figure 12(e1)) compared to the bare membrane (Figure 12(e2)). The untreated membrane exhibited fouling of As^3+^ and As^5+^, which possess an effect on diminishing the adsorption of the membrane by 10% in bovine serum albumin (BSA). The TFN membrane showed an incredible improvement in flux, antifouling, antimicrobial, and antibacterial effects owing to the dramatically reduced pore size of 25 and 35 nm as well. Graphene oxide (GO) was modified with acetic acid (CH_3_COOH), CuFe_2_O_4_, and triethylenetetramine (TETA) on polyethersulfone (PES). Protic ionic liquid (PIL) was used as the support layer. The modified membrane at 0.5 wt % recorded an increase in water flux to 27.87 kg m^−2^ h with an FRR of 91.7%. Owing to the presence of hydrophilic groups such as (-OH, -NH_2_, and -NH), a protective layer was made on the membrane surface, which provided a significant resistance to fouling compared to the bare membrane. The nanocomposite membrane showed a high rejection to monovalent, divalent ions and dyes such as (Methylene blue 94.2%, Crystal violet 95.5 Direct red-1 98%, Reactive black-5 99.5%) [128]. Ethylene glycol is coated with polyaniline (PANI), chitosan, and polyethersulphone (PES) as nanoparticles. The introduction of nanoparticles with their functional groups made the NF membrane more hydrophilic and increased the water flux from 8.04 L m^−2^ h^−1^ to 11.55 L m^−2^ h^−1^ when added to 1 wt% PANI. The rejection rates of Cr^2+^ and Cu^2+^ were 86% and 84%, respectively, using PANI 1 wt%. An increase in the cleaning ability of the antifouling membrane occurred after the addition of the nanocomposite [39]. Iron doped with Al_2_O_3_ to form a nanocomposite (Fe/Al_2_O_3_) was incorporated into polysulfone (PS). The nanocomposite membrane exhibited a marked rejection of Pb^2+^ (99 ± 0.6) and Cd^2+^ (98 ± 1). Antifouling behavior was observed at 10 ppm of both Pb^2+^ and Cd^2+^ and BSA (200 ppm), which decreased after the addition of a nanocomposite membrane. The total porosity and flux increased directly with an increase in the nanocomposite concentration. The relationship between the flux and the applied pressure is shown in Figure 12f, which provides a strong observation of the agreement between increasing pressure and flux; the nanocomposite membrane has a high flux compared to the untreated membrane [129].

It is important to obtain membranes under specific conditions, which require optimized conditions. By describing some operational strategies for membrane fabrication, it is possible to measure the performance of the membrane in the separation of heavy metals. It is important to consider the pH, temperature, concentration of the feed solution, and pressure. In addition, the membrane pore size and fouling resistance during the manufacturing of nanofiltration membranes must be considered.

## 7. Commercialized Nanofiltration Membranes

Membranes are widely used membranes in industries for water treatment of large applications in the removal of salts, dissolved organic matter, and heavy metals. Despite these extensive applications, the addition of nanoparticles to commercial membranes is still under investigation. The addition of nanoparticles in the form of fillers, nanoembedded materials, or nanocomposites has a high cost for commercial availability. Despite the sophisticated ongoing research, new additions of nanomaterials to nanofiltration membranes may be implemented.

### 7.1. Dow Filmtec or DuPont^TM^

It contains a large range of nanofiltration membranes such as NF-90, NF-245, and NF-270. They possess substantial techniques for desalination, purification, and removal of heavy metals. An ideal membrane for surface water, iron, nitrate ions, groundwater, and dissolved organic matter. Some types have molecular weights ˃ 300 amu. Al-Rashdi B et al. [19] examined the commercial NF-90 and NF-270 in the removal of heavy metals and measured their performance compared to commercial RO membranes. NF-90 and NF-270 recorded a higher rejection rate of Pb^2+^, Cr^3+^, Ni^2+^, Cd^2+^, As^3+^, Mn^2+^, and Sb^3+^ than commercialized RO membrane.

### 7.2. GE Water and Process Technologies (SUEZ)

Nanofiltration of the viola provides a membrane with high flux and salt rejection. This is based on the feed concentration and low fouling effect. They help in the extraction of dyes, sulfate, sodium chloride, and metals and in bio-treatment. They produced nanofiltration membranes using a pressure reaching (300 Da). It has several well-known types, such as DK, DL, and HL. Bennani et al. [130] reported the removal of Cd^+2^, Cu^2+^, and Zn^+2^ when the concentration of each heavy metal was fixed at 10^−5^ mol L^−1^ using both DL and DK nanofiltration membranes. The rejection was 86, 90, and 93% for Cd^+2^, Cu^2+^, and Zn^+2^, respectively, for the Dl membrane. However, DL recorded a high flux deviation compared to the pure water lines because of the massive aggregation of ions on its surface.

### 7.3. Hydranautics (Nitto Group)

Nanofiltration membranes have been designed to remove ions, oil, salts, pathogens, pesticides, dyes [131], bacteria, and viruses at molecular weights ranging from 200 to 1500. ESNA and NANO-SW were the most popular types (the Nitto group). Yoon et al. [132] used ESNA-commercialized NF membrane and commercialized RO membrane in the removal of Cr^6+^ and As^5+^. Commercialized RO membranes exhibited better performance than ESNA and measured rejection of >90% for Cr^6+^ and As^5+^, but EANA reached a range of 30–90%.

## 8. Conclusions and Recommendations

The use of nanofiltration membranes is of great importance in membrane separation technology. The integration of nanoparticles onto a nanofiltration membrane surface has a significant impact on the adsorption, selectivity, permeability, and rejection properties of the membrane surface. Nanoparticles are promising solutions for the removal of heavy metals, such as Pb, Cu, Cr, and Zn. Sustainable fabrication of nanofiltration membranes is achieved by phase inversion or interfacial polymerization. Implementation of nanofillers in the form of MWCNTs, PPD, UiO-66-NH_2_, SiO_2_, TiO_2_, and GO in the fabrication of nanoembedded membranes, nanocomposite membranes, and thin-film nanocomposites. These are the best candidates for eliminating contaminants from water. Strategies such as in-situ polymerization, layer-by-layer assembly, blending, coating, and embedding are popular tactics for the elimination of Pb^2+^, Cd^2+^, Co^2+^, and Cu^2+^ by nanofiltration membranes. It was concluded that the introduction of nanomaterials into nanofiltration membranes is a promising way to improve the separation of heavy metals. Future studies should investigate sustainable nanocomposites that can be prepared by incorporating two or more nanomaterials_._ These hybrid materials open a new world in terms of the performance of NF membranes, which is reflected in their better permeability, porosity, diminishing fouling effects, and reduced surface roughness. Nevertheless, operational conditions, such as pH, temperature, concentration, and pressure, must be considered. Furthermore, the membrane pore size and separation techniques are responsible for the selection of heavy metals. Membrane fouling has a significant impact on NF membranes, which must be considered in the commercial synthesis field. Despite the unique characteristics of nanomaterials, further research should explore their effectiveness and potential for heavy metal removal using nanofiltration membranes. Similarly, more environment-friendly and low-cost nanoparticles with outstanding performance in NF membranes are required.

## Figures and Tables

**Table 1:** The maximum contaminant level (MCL) of plenty of heavy metals and their health effects [5].

Heavy Metal	Hazards	MCL (mg L^−1^)
As	Carcinogenic	0.05
Cd	Carcinogenic, headache	0.01
Cr	Carcinogenic, vomiting	0.05
Cu	Carcinogenic, nausea, coughing	0.25
Hg	Diseases of the kidneys, circulatory, and nervous systems	0.00003
Ni	Carcinogenic	0.20
Pb	Diseases of the kidneys, circulatory, and nervous system	0.006
Zn	Damage to the nervous system	0.80

**Table 2:** Summary of various types of nanocomposite materials.

Nanocomposite	Polymers	Heavy Metals	Heavy Metal Rejection (%)	Method of Fabrication	Flux (L m^−2^ h^−1^)	Effect of Addition of Nanocomposites	Ref.
TiO_2_	PA	Cu^2+^, Hg^2+^ and Pb^2+^	87.03 ± 2	IP	-	Increasing the antifouling properties	[13]
TiO_2_/NH_2_	PES	Monovalent, divalent ions	99.7	IP	-	Rising pure water flux	[77]
GO	PPSU	As, Cr, Cd, Pb, and Zn	>98% for anions and ~80% for cations	-	27 ± 3	Rising feed concentration	[78]
GO	PEI	Zn^2+^, Cd^2+^, Cu^2+^, Ni^2+^, and Pb^+2^	97	-	70.3	Increasing antifouling performance with cationic surfactants	[69]
MSNs	PS	Cd^2+^ and Pb^2+^	99	Phase inversion	6.7	Rising mechanical strength hydrophilicity and water flux	[79]
GO/EDA	PA	Zn^2+^, Cu^2+^, Ni^2+^ and Pb^2+^	93.33	IP	18.03	Enhancing the macropores effect on the surface of the hollow fiber composite	[80]
Fe_3_O_4_/SiO_2_	PES	Cd^2+^	93	Phase inversion	65	Modify the stability of the membrane	[81]
SiO_2_ ‘mesoporous’	PS	Cd^2+^ and Zn^2+^	>90	Phase inversion	13 ± 2	Growth of physico-chemical properties	[82]
Cellulose	PA	Cu^2+^ and Pb^2+^	98.4	IP	23.92	Increase nanofiltration membrane flux	[83]
Ag	PA/PEI/PEG	Pb^2+^, Cd^2+^, Co^2+^, and Cu^2+^	>99	IP	40	Reduction of surface pore size	[10]
MWCNTs	PDA/PA	Zn, Mg, and Cu	93.0	IP	15.32	Increasing salt rejection	[84]
MWCNTs/ED	PES	Zn, Mg, Cd, Cu, Ca, Ni, and Pb	96.7	Self-assembly	80.5	Evaluation of thermal and mechanical stabilities	[24]
CNFs/Cs	PES	Cu, Cr, and Pb	98.40	-	13.58	Evaluation of surface hydrophilicity	[85]
Mil-125(Ti)/CS	PES	-	-	Phase inversion	-	Increment of antifouling properties	[86]
FeS/CFFO	PVDF	Cr^6+^, Cd^2+^, and Pb^2+^	99	Phase inversion	340–1266	Rising the water flux, porosity, and hydrophilicity	[87]
ZnO/FeOOH	PET	Pb^2+^ and Cr^6+^	94.7	Electro-spinning	169.3	Better antifouling properties	[88]
F-CMK-5	PES	Zn^2+^ and Fe^2+^	-	Phase inversion	-	Recording a dramatic increment in heavy metal rejection	[89]
GO	PES	Cu, Zn, and Cd	>80	Phase inversion	∼55	Rising the salt, dye, and heavy metal rejection	[90]

Abbreviations. PA (polyamide), IP (interfacial polymerization), PS (polysulfone), PES (polyethersulfone), PPSU (polyphenylsulfone), PEI (polyethylenimine), MSNs (Meso porous silica nanocomposite), EDA (Ethylene diamine), PEG (polyethylene glycol), PAN (Polyacrylonitrile), MWCNTs (Multi-walled carbon nanotubes), ED (Ethylenediamine), CNFs (Carbon nanofibers), CS (chitosan), FeS (Ferrous sulfide), CFFO (Carboxyl-functionalized ferroferric oxide), PVDF (polyvinylidene difluoride), PET (polyethylene terephthalate), F-CMK-5 (functionalized mesoporous carbon).

**Table 3:** Some NF membrane modifications for reducing membrane fouling.

Mitigation Fouling Process	Fouling Substrate	Treatment	Nano Particle	Efficiency	Adv.	Flux	Ref.
Pre-treatment of feed solution	1,3-propanediol broths in feed,	NF270	-	97% MgSO_4_	Complete removal of Fe, S, Si, C, Al, P, and Ca deposits and bacterial fermentation fouling	0.18 L m^−2^ h^−1^	[119]
Grafting	*E. coli*	PSF	GO/Pt 0.75 wt%	24% NO_3_^−^	Rising the membrane hydrophilicity	675.71 L m^−2^ min^−1^	[120]
Grafting	Hydrophobic fouling	Thiolated zwitterionic polyurethane 30 g L^−1^/PDA/PES	-	95% NaCl	High fouling resistance compared to commercially benchmark NF membrane	50 L m^−2^ h^−1^	[121]
Grafting	Humic acid and Congo red	Zwitterion	-	Humic acid 98.1% and Congo red 97.6%	Removal of organic pollutants	-	[122]
Coating	Dye and heavy metals	PVDF	SiO_2_^+^ PEI	74.2% Cu^2+^	Rising the anti-fouling ability	10,700.0 ± 353.3 L m^−2^ h^−1^	[123]
Cross-linking	Biological source	Tannic acid/polyvinylamine	-	>99% for Ca^2+^ and Mg^2+^	The permeability improved by 60%	-	[124]
Blending of polymeric matrix and embedding of nanoparticle	Protein	PES/TPU	GO-APTS	99.4% methylene blue	Rising FRR to 92.9%	∼74 L m^−2^ h^−1^	[117]

Abbreviations. NF270 (Nanofiltration 270), PSF (polysulfone), PVDF (Polyvinylidenedifuloride), PEI (Polyetherimide), PES (polyethersulfone), TPU (Thermoplastic polyurethane), APTS ((3-aminopropyl) triethoxysilan), FRR (flux recovery ration).
